# Middle Ear Transducer: Long Term Stability of the Latest Generation T2

**DOI:** 10.1155/2019/4346325

**Published:** 2019-01-06

**Authors:** Nils Kristian Prenzler, Eugen Kludt, Thomas Giere, Rolf Salcher, Thomas Lenarz, Hannes Maier

**Affiliations:** ^1^Cluster of Excellence Hearing4all, Germany; ^2^Department of Otolaryngology, Hannover Medical School, Hannover, Germany

## Abstract

**Objectives/Hypothesis:**

Comparing long term stability of the Middle Ear Transducers (MET) of the 1st generation T1 (Otologics LLC) with the current generation T2 (Cochlear Ltd.) in all our clinical cases with standard incus coupling.

**Study Design:**

Retrospective chart review.

**Methods:**

52 ears implanted with a MET device between 2008 and 2016 were analyzed retrospectively. All patients suffered from sensorineural hearing loss and the actuator was coupled to the body of the incus (standard coupling). 23 ears were implanted with the transducer T1 (Otologics LLC) between 2008 and 2011 and 29 ears were implanted with the current transducer T2 since 2011 (Otologics LLC/Cochlear Ltd.). Latest available in situ and bone conduction (BC) thresholds were exploited for a follow-up period of up to 7 years after first fitting. Long term stability of coupling and actuator performance was evaluated by tracking differences between in situ and BC thresholds.

**Results:**

In the T1 group, 9 out of 23 implants were still used by the patients at their last follow-up visit (average observation time 3.7 yrs.; min 1.0 yrs., max 7.4 yrs.). In 9 patients a technical failure identified by a decrease of in situ threshold of more than 15 dB compared to BC thresholds [Δ (in situ – BC)] lead to non-usage of the implant and 7 explantations. Five other explantations occurred due to medical reasons such as BC threshold decrease, infection, or insufficient speech intelligibility with the device. In the T2 group, 23 out of 29 implants were still used at the most current follow-up visit (average observation time 3.3 yrs.; min 1.0 yrs., max 4.8 yrs.). No technical failures were observed up to more than 4 years after implantation. Five T2 patients discontinued using the device due to insufficient benefit; two of these patients were explanted. One patient had to be explanted before the activation of the device due to disorders of wound healing. Nevertheless, a small but significant decrease of hearing loss corrected coupling efficiency [Δ (in situ – BC)] was seen in the T2 group.

**Conclusions:**

In contrast to the T1 transducers of the earlier generation of MET systems where technical failures occurred frequently, no technical failures were detected after 29 implantations with the current T2 transducers. However, a small but significant decline of transmission efficiency was observable even in the T2 implanted group.

## 1. Introduction

The partially implantable active Middle Ear Implant (AMEI) called the Middle Ear Transducer (MET, Cochlear Ltd.) consists of an external audio processor and an internal implant. The implant comprises the transmission coil, the demodulator, and an electromagnetic actuator that is mounted into mastoid with a fixation bracket. In the standard application in patients with intact ossicular chain and moderate-to-severe sensorineural hearing loss (SNHL), the tip of the transducer is attached to the body of the incus and converts the electric signal to mechanical vibration. The same stimulation principle and transducer are used in the current fully implantable device Carina™ (Cochlear Ltd.).

The MET system was described by Kasic in 2001 [1] and first data regarding the audiological outcome was published by Jenkins et al. in 2004 [2]. Patients achieved equal or better results in speech recognition compared to best fitted conventional hearing aids (HAs). Since then, the implant has been widely used especially in patients with contraindications for conventional HA, e.g., recurrent external otitis with effusion.

Over the course of time, more and more data emerged that constituted frequent technical failure of the device. Zwartenkot et al. [3] reported on a technical failure rate of 9/32 over a follow-up period of up to 13 years, whereby all failures occurred within the first 2 years after implantation. Four of the 32 patients in that publication had been implanted with a Cochlear® MET (T2) since 2014, where no failure occurred so far, insinuating an improved technical long term stability of the T2 transducer compared to the T1.

The MET and Carina systems successfully fill an important gap in commercially available AMEIs for the treatment of SNHL as the audiological indication range exceeds the one of Vibrant Soundbridge (VSB) [4] and bone conduction implants [5–7]. In contrast, the direct acoustic cochlear stimulator (Codacs ®, Cochlea Ltd.) was a powerful system that was intended only for mixed hearing loss, in particular for cases of otosclerosis [8], but is not on the market anymore.

The aim of the present study was to investigate the long term stability of the new generation of T2 transducers of the MET system as a stable coupling efficiency over time and technical reliability of the actuator are crucial for further recommendation of this implant to patients.

## 2. Methods

A retrospective cohort study was performed at a tertiary university hospital. All patients implanted with the MET system between 2008 and 2016 with standard coupling were included in the study. The surgical procedure was performed as described before [2] and all patients fulfilled audiological indication criteria as recommended by the manufacturer. Twenty-three MET T1 systems (Otologics LLC) were implanted in 18 patients. Starting in 2011, 29 MET T2 systems (Otologics LLC and Cochlear Ltd.) were implanted in 27 patients. Both groups are analyzed here.

Patients were counted as user or non-user depending on their response at their most current visit to the clinic when asked if they still use the device. To our knowledge 4 patients discontinued routine visits to the clinic due to death. Cases of non-usage and explantations were defined as implant losses. The “implant loss” group was divided further into medical and technical reasons for non-use, depending on the decrease of stimulation efficiency. As criterion for technical failure a decrease of the* in situ* threshold > 15 dB relative to the BC threshold was used [Δ (*in situ* – BC)] (see Indicator for Actuator Performance and Coupling Efficiency). This in turn implies that a decrease in coupling efficiency of > 15 dB alone in a patient that still uses his implant successfully was not rated as technical failure. All remaining patients in the implant loss group that did not fulfill the criterion for technical failure were rated as medical reasons. No further distinction between explantations due to medical problems, e.g., infections or non-usage due to insufficient audiological benefit, was made in this group.

### 2.1. Reference Transmitter vs. BAP 2 Speech Processor

**Figure 1:**
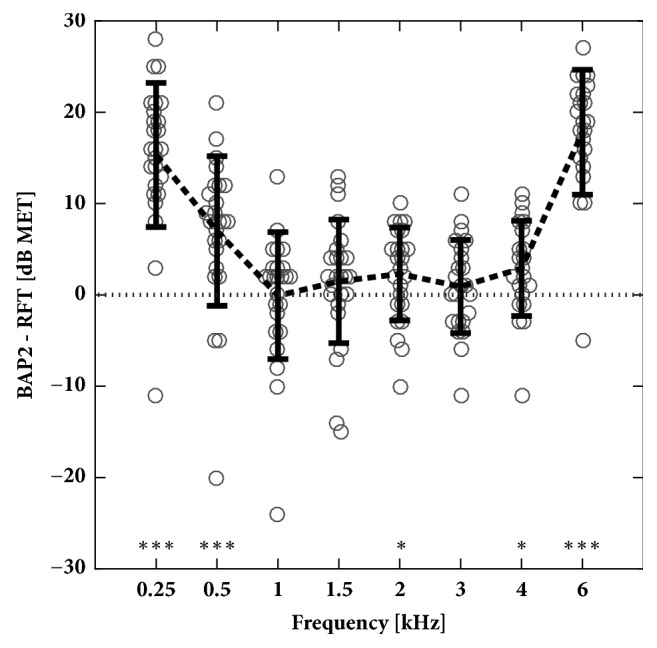
Differences of in situ thresholds determined with the BAP 2 audio processor and the reference transmitter (RFT). In situ thresholds determined with the BAP 2 were usually higher, especially at low and high frequencies where the difference was statistical significant. Differences were normally distributed at all frequencies (Kolmogorov-Smirnov test). Statistical difference between RFT and BAP 2 measurements was evaluated with the Student t-test (*∗* p < 0.05; *∗∗∗* p < 0.001). Individual results are shown as circles and means as a dashed line, and error bars show standard deviations.

Until 2011,* in situ* thresholds were measured with a separate device called the reference transmitter (RFT), but since then,* in situ* thresholds were measured directly with the Button Audio Processor BAP 2. The reference transmitter is not used nor supported by the current manufacturer. In order to make “old” (reference transmitter) and “new” (BAP 2) data comparable, 25 implants (23 patients) were measured with both methods on the same day and the differences between the two strategies (BAP 2 - RFT) were averaged for 0.25, 0.5, 1, 1.5, 2, 3, 4, and 6 kHz (Figure 1; Table 1). By this, all results obtained earlier with the reference transmitter were converted to make them comparable to BAP 2 results measured later. All results shown in the following analysis are equivalent to* in situ* thresholds determined with the BAP 2.

### 2.2. Bone Conduction Threshold

Pure tone averages (PTA4) for BC and* in situ* (BAP 2) thresholds were calculated from respective thresholds at 0.5, 1, 2, and 4 kHz. In our analysis patients were evaluated only where complete BC threshold data for all relevant time points was available. BC thresholds that were below the limits of our audiometers were excluded, except in cases with no relevant air-bone-gap (ABG) after surgery. Only in cases where the ABG was ⩽ 10 dB at first activation of the implant, AC thresholds were used as estimate for BC thresholds when a BC threshold was outside the limits of the audiometric equipment. If the ABG was higher, no estimation was performed and values for these frequencies were omitted.

The difference Δ BC was calculated as the difference between BC PTA4 at the latest measurement (BC_last_) and the preoperative measurement (BC_pre-op_) to track the evolution of the individual SNHL after surgery over time. Negative values indicate a progression of hearing loss (Δ BC = BC_pre-op_ – BC_last_).

### 2.3. Indicator for Actuator Performance and Coupling Efficiency

The difference (*in situ* – BC) was calculated as the difference between* in situ* and BC PTA4s at the first fitting and the latest available measurement after implantation to track the performance of the actuator and the efficacy of transmission to the ossicles independently of the progression of SNHL. This “coupling efficiency” will be in arbitrary units as* in situ* thresholds are device specific and not calibrated and will depend on the individual coupling situation to the ossicles. As individual differences depend on the initial coupling efficiency, results for each patient can be used to track the transmission to the ossicles over time, when referenced to the (*in situ* – BC)_firstfit_ obtained at activation. Here we used the difference Δ (*in situ* – BC) = (*in situ* – BC)_firstfit_ - (*in situ* – BC)_last_ that is independent of the initial coupling efficiency and progression of sensorineural hearing loss. Using this definition, negative values indicate a decrease of either coupling efficiency or actuator performance. Although all patients had a measurable* in situ* threshold initially, some patients left the range where* in situ* thresholds could be technically determined. This required a well-defined estimation procedure. In cases when no* in situ* threshold was measurable, the device limit plus 1 dB was used to estimate the* in situ* threshold at the latest visit. Limiting the* in situ*_last_ threshold at the last visit leads to a best case estimate of the coupling efficiency Δ (*in situ* – BC) which was used in the following analysis.

### 2.4. Statistical Analysis

Statistical comparison was performed in Matlab R2016a (the MathWorks, Inc., Natick, Massachusetts, United States.), using the Kolmogorov-Smirnov test and Student t-test from the Statistics Toolbox, Kaplan-Meier estimation for implant survival as well as log-rank (Mantel-Cox) test from Matlab Exchange [9, 10]. Differences were considered statistically significant if p < 0.05.

## 3. Results

### 3.1. Patients and Demographics

A total of 43 patients implanted between 2008 and November 2016 with 52 MET implants at our institution were analyzed retrospectively (19 males, 24 females, mean age at implantation: 61, range 24 to 83 years). Two of these patients were re-implanted with a T2 transducer after failure of the T1 and contributed data to the T1 and T2 group. Beside one explantation due to ongoing disorder of wound healing, no further major complications were reported perioperatively.

In the T1 group, 9 out of 23 implants were still used by the patients at their last follow-up visit. In 9 implants a technical failure, identified by a decrease of* in situ* threshold of more than 15 dB (or beyond measurement limits) compared to BC thresholds, led to non-usage of the implant and 7 explantations. Five other implant losses occurred due to medical reasons such as BC decrease, infection, or insufficient speech intelligibility with the device. In the T2 group, 23 out of 29 implants were still in use at the last follow-up visit. Here no technical failures were observed up to more than 4 years after implantation. Five patients discontinued using the device due to insufficient benefit with the device. Two of these patients were explanted. One patient had to be explanted before the activation of the device due to wound healing disorders. In summary the average observation time of transducer performance in the T1 group was 3.7 yrs. (min 1.0 yrs., max 7.4 yrs.) and in the T2 group 3.3 yrs. (min 1.0 yrs., max 4.8 yrs.). In total 74.7 patient years (T1) and 78.2 patient years (T2) were available for our analysis presented here.

### 3.2. Bone Conduction Thresholds

**Figure 2:**
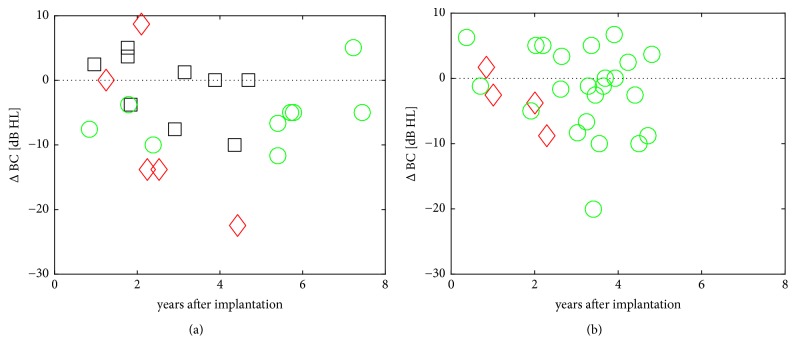
Change in BC threshold (Δ BC) relative to pre-op over time for (a) the T1 group, (b) the T2 group. Symbols: (green circle) users, (black square) non-users and explantations due to technical failure, (red diamond) non-users and explantations due to medical reasons. No significant difference in Δ BC between the T1 and T2 group (two sample t-test) was found.

The changes Δ BC in the T1 group between first fitting and the latest available result were between +8.8 dB and – 22.5 dB HL (Figure 2(a)). As expected, the longer the follow-up lasted, the higher the hearing losses were. Accordingly, Δ BC in the T2 group changed between first fitting and the latest available data between + 6.7 and – 20.0 dB (Figure 2(b)). The average sensorineural hearing loss per patient year in BC thresholds was similar in both groups (group T1: −1.0 ± 2.7 dB HL/yr.; group T2: −0.9 ± 2.1 dB HL/yr.; mean ± SD.

### 3.3. Actuator Coupling Efficiency

**Figure 3:**
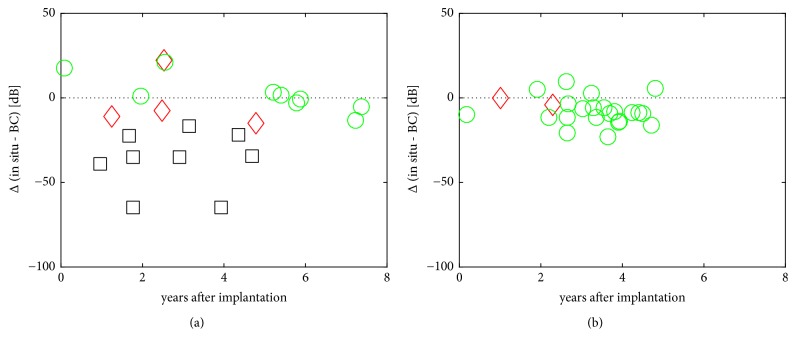
Actuator performance/coupling efficiency as Δ (in situ – BC) for (a) the T1 group and (b) the T2 group. Symbols: (green circle) users, (black square) non-users and explantations due to technical failure, (red diamond) non-users and explantations due to medical reasons. T1: N = 22, T2: N=27 (in situ thresholds from one T1 and two T2 subjects were missing).

In the T1 group the difference between* in situ* and BC thresholds Δ (*in situ* – BC) (Figure 3) dropped by – 65 dB in the worst case. When technical failures were excluded, the mean difference in the remaining cases was Δ (*in situ* – BC) = 0 ± 4.8 dB per patient year (mean ± SD). A statistical analysis in this group was not performed as it would supposedly be distorted by the numerous implant failures/losses.

Regarding the differences between* in situ* and BC thresholds in the T2 group, the mean decrease over all cases was Δ (*in situ* – BC) = -2.1 ± 2.7 dB per patient year (mean ± SD; t-test: statistically significant different from 0 with p < 0.001).

**Figure 4:**
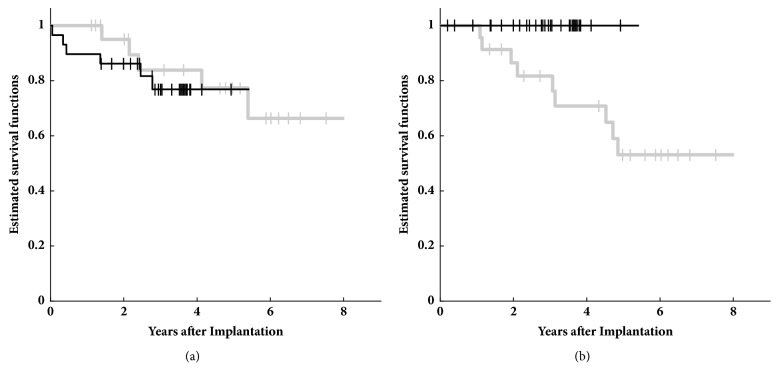
Kaplan-Meyer plot for of implant survival for T1 (grey) and T2 (black) transducers. Explantations or non-usage due to medical reasons is presented in subfigure (a) and explantations or non-usage classified as technical failures is presented in subfigure (b). Differences between T1 and T2 survival rates were only statistical significant (p < 0.001) for technical failures.

Kaplan-Meier curves for implant survival were calculated separately for medical and technical reasons (Figure 4). The log-rank test indicated no statistically significant difference between T1 and T2 regarding implant losses due to medical reasons. Looking at implant failure due to technical reasons, T1 transducers had a median survival time of 4.8 years while no T2 transducers failed in the observed period of time. The log-rank test indicated a significant difference (p < 0.001) between the corresponding Kaplan-Meier estimates.

## 4. Discussion

This article reports on the reliability of the MET T1 and MET T2 transducer generation in terms of efficacy and stability in the standard incus coupling application. To our knowledge this large collective of new generation MET T2 systems covers the longest follow-up period in literature so far.

The MET transducer is used in two different devices: the semi-implantable MET that is commercially not available anymore and the fully implantable device Carina®. The rare articles that report on the semi-implantable MET state technical problems of the earlier MET T1 generation but only few cases are mentioned regarding the latest generation devices [3, 7]. Debeaupte et al. [11] make a clear distinction between different generations of Carina implants and report on implant survival after 2 years in a multicenter setting. In this publication, devices with a T1 first generations transducer showed failures in a significant fraction (32%; 30/95) in contrast to implants with T2 transducer (5%; 2/42) 2 years after implantation. Studies analyzing the survival of the MET T1 over longer terms indicate either lower (16% [6]) or similar high failure rates (28% [3]). However, studying the survival rate of the transducers separately is difficult or impossible in Carina® implants as the device was prone to other sources of defects (battery, connectors) than failures of the transducer or loss of coupling efficiency. For this reason we investigated implant survival in MET devices separately for T1 and T2 generation transducers over an extended time period.

Constancy of the so-called* in situ* threshold determined via the implant indicates the integrity of the device, the transducer, and unchanged coupling efficiency to the ossicles. However, it underlies the individual hearing loss and* in situ* thresholds have to be compensated for loss in inner ear function. Hence the difference between* in situ* and BC thresholds Δ (in situ – BC) can be assumed a good indicator for stable coupling efficiency and integrity of the device. To cover a sufficiently long observation time, it was necessary to translate* in situ *measurements with the former reference transmitter into values from measurements with the later BAP 2 system. This issue was solved by performing measurements with both techniques in patients. Doing so, earlier data from patients were comparable to more current data obtained with the BAP 2.

Our evaluation documents the failure of many MET T1 devices over the first years after implantation with a similar failure rate described by Zwartenkot et al. [3]. Looking at individual cases, we commonly saw a slow increase of Δ (*in situ* – BC) rather than an abrupt loss of device function (data not shown).

In 2011, the T2 transducer was brought onto the market and since we implanted this device, no technical failure occurred so far in at least 28 cases (one explantation before activation was excluded) covering an observation time of up to 4 years. Analyzing all our T2 cases, we saw a small but statistically significant increase of the difference between* in situ* and bone conduction thresholds [Δ (*in situ* – BC)]. Our reference of coupling efficiency was at activation early; hence early effects within the first weeks could not have contributed to our results. As our patients usually come in for routine visits once a year, the broad spacing of available results does not allow a detailed analysis of the time course. A visualization of the coupling efficiency over time with the available data gave no hints to differentiate between a continuous drop or event-like changes. Hence we only can speculate about the possible reasons for the decreasing coupling efficiency over the years after implantation. (1) The transducer is connected to the incus with a certain preload. Over time, the ligaments that attach the ossicles to the tympanic walls might yield and thereby attenuate the preload that is necessary for sufficient transmission of power. (2) The tip of the transducer may slip away from the initial position at the incus, possibly induced by pressure changes in the tympanic cavity or by remodeling processes of the ossicle due to small but chronically applied pressure. (3) Increased stiffness of the ossicular chain could be caused by non-physiologic generated vibration patterns during the course of years or by sclerosis or fibrous tissue around the transducer or the ossicular chain as a consequence of the surgical procedure. However, it is unlikely that the actuator performance is reduced by tissue growth as demonstrated previously [12].

Nevertheless, also technical sources such as a minimal loss of power of the T2 transducer, although unlikely, cannot be excluded with the present approach, as measuring the implant function (transducer* and* coupling efficiency) by Δ (*in situ* – BC) does not disclose the origin of possible causes (transducer* or* coupling efficiency). Further (experimental) investigations are needed to elucidate both the source and the development of the small decrease of implant function in the T2 group.

In the T1 collective, in contrast, we found a massive decrease of Δ (*in situ* – BC) in most of the cases indicating that either the coupling or the transducer function was disturbed. As we found only a slight decrease of Δ (*in situ* – BC) over years in the T2 group, it is most likely that a loss of the transducers function led to progressive failures in the T1 group as the coupling method was identical with the T2 group.

## 5. Conclusion

Throughout an observation period of up to 4 years, we observed no technical failures when using the latest generation of MET systems with T2 transducers in contrast to the earlier generation T1 (average observation time: T1 3.7 yrs. (min 1.0 yrs., max 7.4 yrs.); T2 3.3 yrs. (min 1.0 yrs., max 4.8 yrs.) covering 74.7 patient years (T1) and 78.2 patient years (T2). However, there was a small but significant decline of stimulation efficiency over that period of time even in the T2 group. Proper follow-up of these patients is mandatory to assess long term stability of this implant system.

## Figures and Tables

**Table 1:** Difference in in situ thresholds determined with the BAP 2 processor and the reference transmitter.

Frequency [kHz]	0.25	0.5	1	1.5	2.0	3.0	4.0	6.0
Mean [dB]	15.3	7.0	-0.1	1.5	2.3	0.9	2.9	17.8
Standard Deviation [dB]	7.9	8.2	7.0	6.8	5.1	5.1	5.2	6.8

## Data Availability

The data used to support the findings of this study are available from the corresponding author upon request.
